# Necrotizing Fasciitis Associated With Toxic Shock Syndrome

**DOI:** 10.7759/cureus.55807

**Published:** 2024-03-08

**Authors:** Shahriar Sharif, Samyukta Swaminath, Nashit Mozumder, Kenneth A Mack, Diego Marin

**Affiliations:** 1 Internal Medicine, HCA Florida Westside Hospital, Plantation, USA; 2 Osteopathic Medicine, Nova Southeastern University Dr. Kiran C. Patel College of Osteopathic Medicine, Fort Lauderdale, USA; 3 Critical Care Medicine, HCA Florida Westside Hospital, Plantation, USA

**Keywords:** necrotizing fasciitis with toxic shock syndrome, modified lrinec score, lrinec score, debridement, septic shock, group a strep, streptococcus pyogenes, toxic shock syndrome, necrotising fasciitis, necrotizing fasciitis

## Abstract

Necrotizing fasciitis is a rapidly progressing bacterial infection that affects the deep fascia and subcutaneous tissues, often resulting in tissue necrosis and systemic toxicity. This case involves a male in his late forties who initially sought emergency care for a minor rash on his right lower extremity and symptoms of a viral illness. Despite an initial diagnosis of hematoma, his symptoms rapidly escalated within 24 hours, prompting his return to the emergency room. During this subsequent visit, signs of septic shock emerged, accompanied by a worsening rash and blister formation.

Admitted to the intensive care unit, our patient received urgent treatment, including broad-spectrum antibiotics and surgical debridement based on the Laboratory Risk Indicator for Necrotizing Fasciitis (LRINEC) score for assessing necrotizing fasciitis severity. Further debridement and a fasciotomy were performed, leading to improved clinical conditions, stabilized vitals, and normalized laboratory results. This case underscores the critical importance of early clinical suspicion, prompt diagnosis, and a collaborative, team-based approach in successfully managing necrotizing fasciitis.

## Introduction

Necrotizing fasciitis (NF) is a rapidly progressing, life-threatening soft tissue infection characterized by rapid necrosis of fascial planes and surrounding structures. The yearly occurrence of necrotizing fasciitis varies, with rates ranging from 15.5 cases per 100,000 population in Thailand to 0.3 to 5 cases per 100,000 in other geographical areas. This condition poses a significant clinical challenge due to its aggressive nature, leading to high morbidity and mortality rates despite advancements in medical care [1].

NF is commonly triggered by polymicrobial or monomicrobial infections, with Group A Streptococcus and Staphylococcus aureus frequently identified as primary causative agents [2]. The infection typically originates from a breach in the skin, such as wounds, surgical incisions, or even minor abrasions, allowing opportunistic pathogens to invade deep fascial layers [3].

Early diagnosis and intervention are crucial for improving outcomes in NF cases. The disease progresses rapidly, and delayed treatment can result in widespread tissue destruction and systemic complications. Various risk factors contribute to the development of NF, including advanced age, immunocompromised states, diabetes mellitus, and chronic medical conditions [4].

## Case presentation

A 46-year-old male with an unremarkable medical history presented to the emergency department with a rash and progressive pain in his right lower extremity. He was initially evaluated in the emergency department for feelings of malaise, mild abdominal pain, myalgias, and chills for about two to three days. He was managing his symptoms with acetaminophen and ibuprofen as needed. An abdominal ultrasound was conducted at that time due to mild transaminitis, but no abnormalities were detected. He was discharged with simethicone and famotidine for a suspected viral illness.

**Figure 1:**
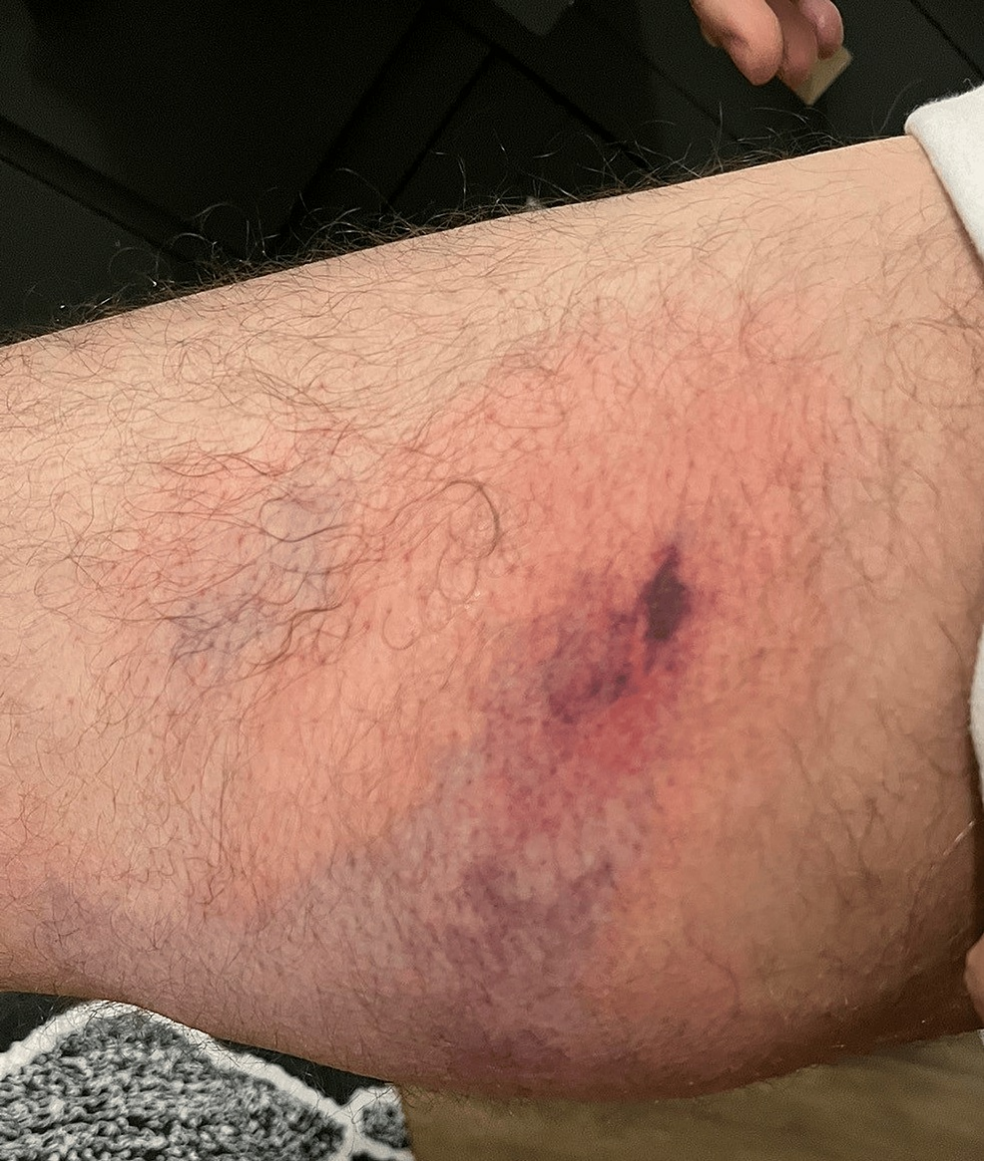
Initial presentation: a small circular, purple rash with surrounding redness, resembling a hematoma.

Shortly after being discharged, the patient noticed an enlarging, painful rash in his right groin area. Concerned, he returned to the emergency room. During this second visit, examination revealed right groin pain and the presence of a small circular, purple rash with surrounding redness, resembling a hematoma (Figure 1). An ultrasound of the lower extremities ruled out deep vein thrombosis, and the patient was discharged with pain medication for the suspected hematoma.

**Figure 2:**
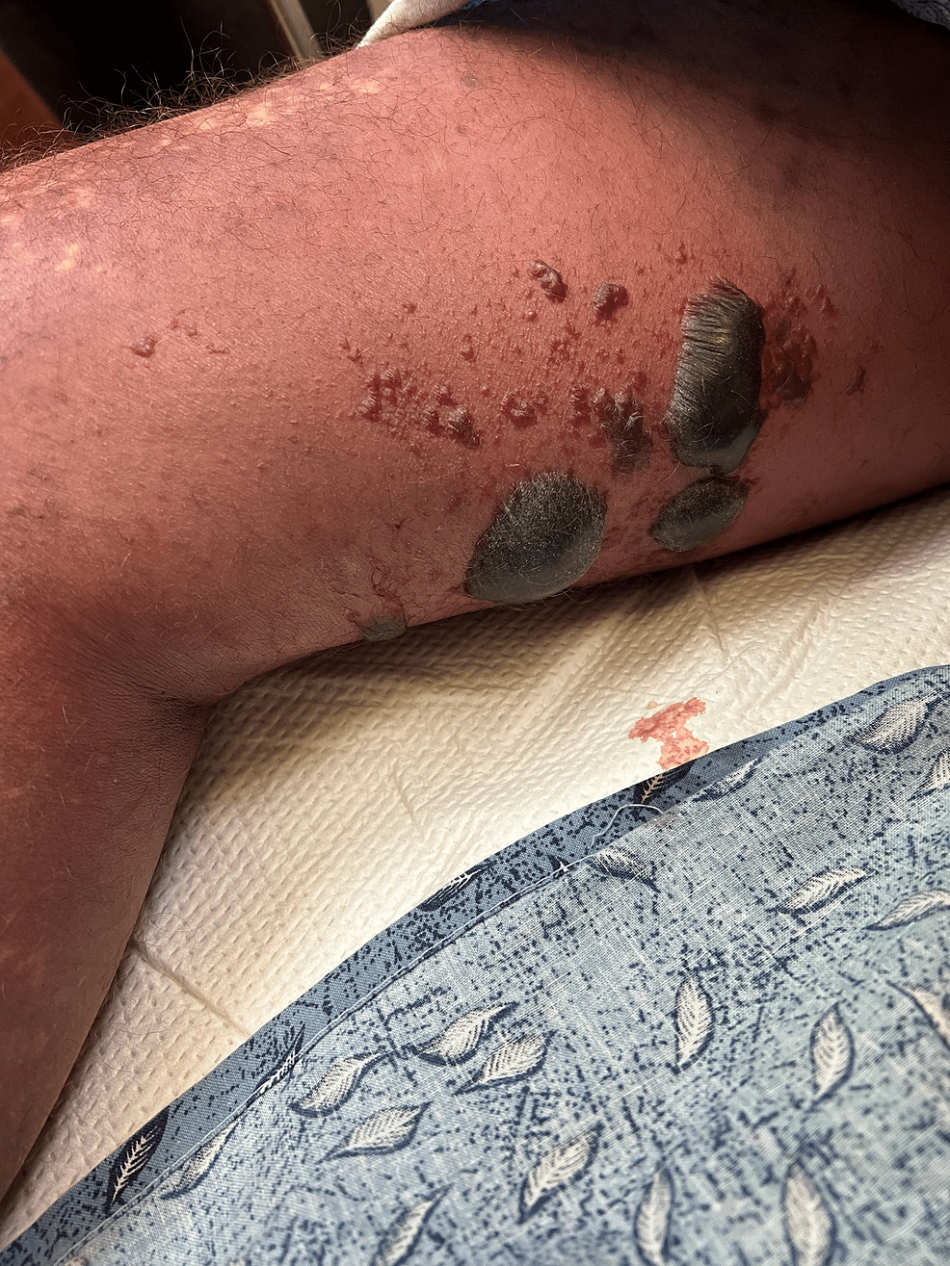
Hemorrhagic blister formation upon subsequent visit.

Within 24 hours of the last emergency room visit, the patient's condition deteriorated. He developed worsening pain, and the rash spread further, accompanied by hemorrhagic blisters (Figure 2). Furthermore, he experienced symptoms of nausea, vomiting, diarrhea, as well as tachycardia, tachypnea, and fever. The patient was hypotensive upon evaluation, raising suspicion for septic shock.

Reassessment revealed a fever (maximum temperature of 100.4 °F) along with a tender, erythematous rash on the right leg with poorly defined margins and a purple center initially free of blisters (Figure 2). He presented with hypotension, recording a blood pressure of 80/50 mmHg, and laboratory findings revealed reduced serum sodium levels along with elevated levels of creatinine, C-reactive protein, total bilirubin, conjugated bilirubin, aspartate aminotransferase (AST), alanine transaminase (ALT), and lactic acid. Subsequently, a non-contrast CT scan revealed cellulitis in the right lower extremity, without evidence of bullae or soft tissue gas at the time. Empiric antibiotic treatment with clindamycin, piperacillin-tazobactam, and vancomycin was promptly initiated. The calculated Laboratory Risk Indicator for Necrotizing Fasciitis (LRINEC) score at the time of arrival was 8, further supporting the diagnosis of necrotizing fasciitis. As a result, the surgical team was urgently consulted for emergent debridement.

Management

**Figure 3:**
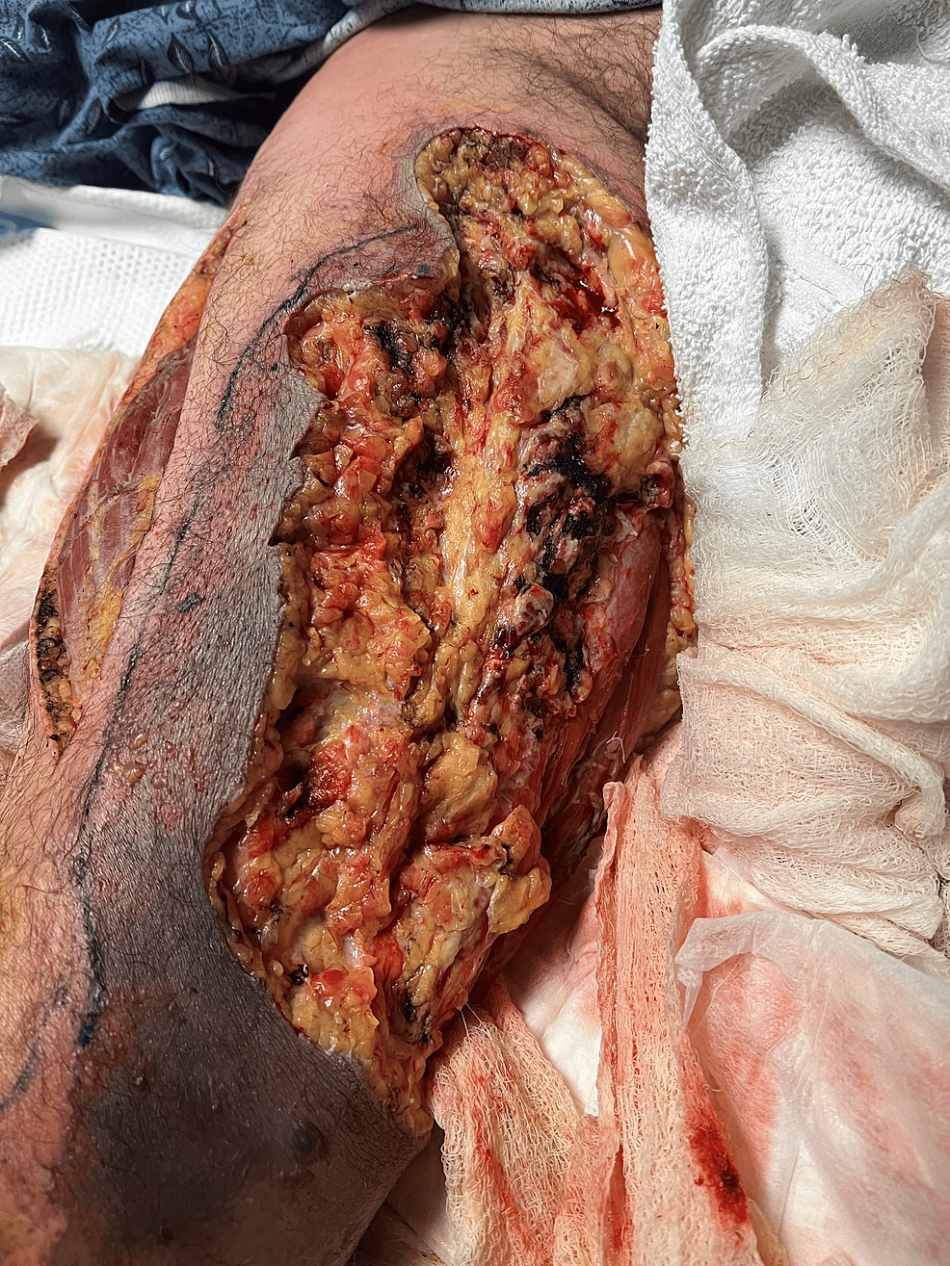
Decompressive fasciotomy and debridement.

**Figure 4:**
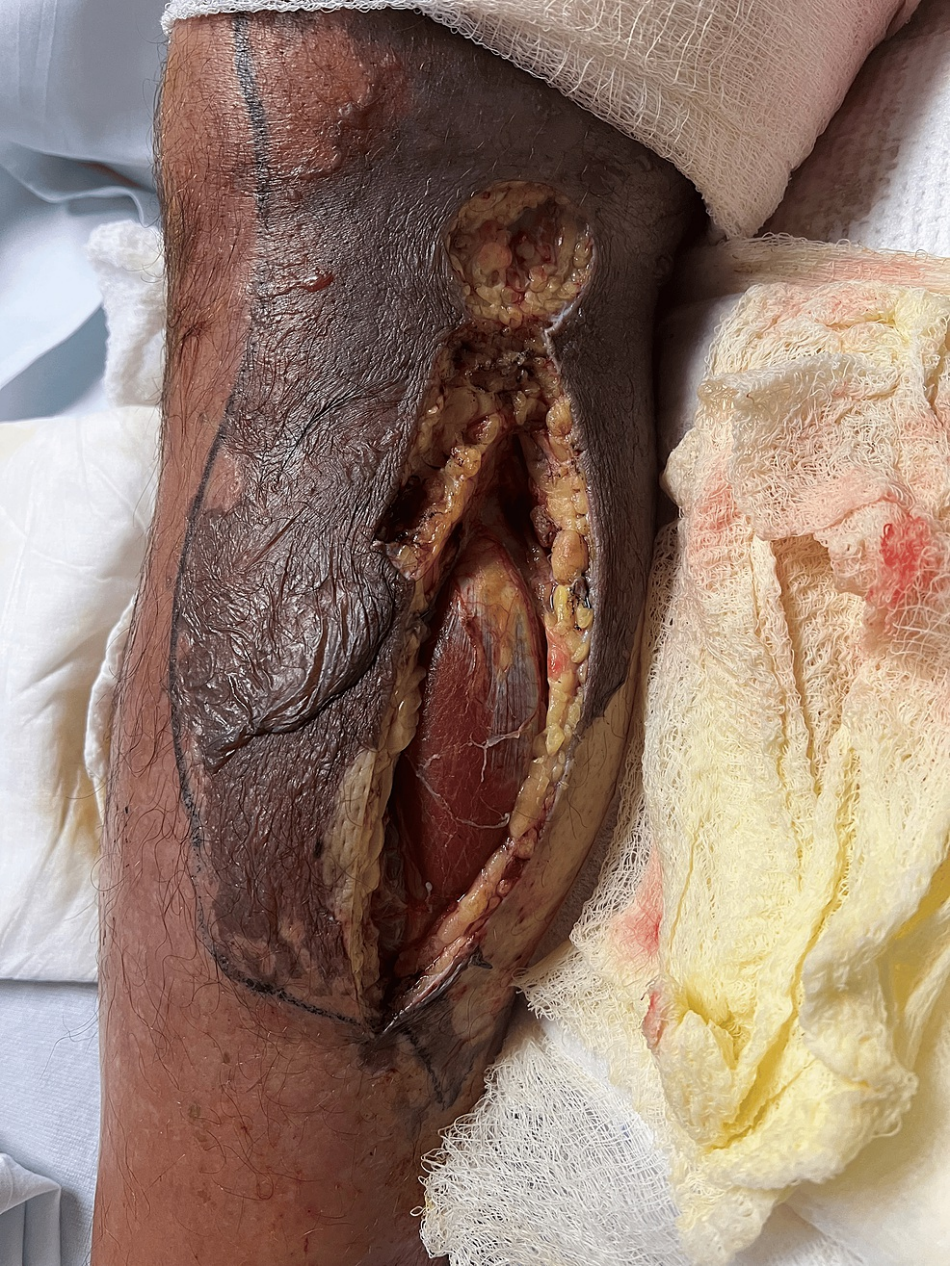
Decompressive fasciotomy and debridement showing intact and viable muscle tissue.

Following surgical evaluation, the patient was promptly brought to the operating room for debridement of the right medial thigh. Subsequently, he underwent repeated incision and drainage on the following day. On the third day of admission, additional decompressive fasciotomy and debridement of the right thigh and leg were performed (Figure 3). On days 7 and 10, the patient underwent repeated excisional debridement procedures to further eliminate affected skin, subcutaneous tissue, and fascia, amounting to five debridement interventions over the 2-week period. Notably, despite fascial involvement, the surgeon observed minimal to no involvement of the muscle or deeper structures (Figure 4).

The patient's antibiotic regimen was changed to intravenous (IV) meropenem, IV vancomycin, and IV clindamycin with guidance from Infectious Disease. On the third day of admission, blood culture and intraoperative cultures confirmed the presence of *Streptococcus pyogenes*, which led to the tailoring of the antibiotic treatment to IV clindamycin and penicillin G.

Outcome and follow-up

After the surgical interventions, the patient exhibited positive signs of recovery, with stable vital signs, improved urine output, and a decrease in white blood cell count. Laboratory results also returned to normal levels. Given his improved clinical state, the patient was transferred to another facility for further treatment including skin graft and plastic surgery reconstruction of the debrided area.

During the follow-up period, the patient underwent an additional debridement procedure and was prescribed a wound vac for five weeks before proceeding with plastic surgery reconstruction using a skin graft. Thanks to the timely diagnosis and multidisciplinary approach, the patient had a positive outcome despite his critical condition, emphasizing the importance of a comprehensive and prompt medical response.

## Discussion

Necrotizing fasciitis is a rapidly developing, potentially fatal bacterial infection that affects the superficial and deep fascia as well as subcutaneous fat, with a mortality rate ranging from 19% to 30% globally despite modern management modalities [5]. It can cause tissue death and subsequent multiorgan failure. Although group A Streptococcus is usually the culprit, Staphylococcus aureus and Clostridium perfringens can also cause NF. The presence of gas and necrotic tissue in imaging is a characteristic feature of NF, although it may not be consistently observed [1].

The pathogens can enter the body through various skin conditions, including deep wounds and insect bites. However, because earlier skin issues are frequently not visible, it is thought that bacteria migrate through the blood. After entering the circulation or as a result of a penetrating injury, the infection typically begins at the site of a muscle injury. The pathogenesis is theorized to be due to virulence factors that allow preferential binding of bacteria to vimentin on the surface of connective tissue proximal to myocytes [6].

NF can be categorized into type 1 and type 2, meaning polymicrobial vs. monomicrobial respectively. Type 1, which accounts for 70-90% of cases, is caused by various organisms such as streptococcal and staphylococcal species. Type 2 is primarily associated with group A streptococci, responsible for about 10% of cases [6].

Risk factors for NF include advanced age, history of chronic infectious diseases, immunocompromised status, diabetes, cancer, and liver disease. It also occurs more frequently in people who have recently undergone surgery, suffered an injury, or experienced trauma to the affected area [2].

The diagnosis of NF is primarily based on history and physical examination, supported by laboratory values and imaging. The initial stages of necrotizing fasciitis are often easily missed due to the absence of specific clinical features, leading to confusion with other skin and subcutaneous infections. Common symptoms include local signs such as redness, swelling, blistering with clear fluid, tissue hardening, bluish-dark skin discoloration, hemorrhagic bullae, and skin necrosis. Additionally, in the early stages, the patient may experience severe pain that is disproportionate to the physical examination findings. As the disease progresses, virulent organisms and toxins are released into the bloodstream from the infected tissue, triggering a systemic toxic reaction [7]. Leukocytosis, elevated inflammatory markers, and acidosis are common lab abnormalities. MRI and CT scans of the affected areas can reveal the extent of the infection by demonstrating soft tissue edema, gas formation, and thickening of the fascia [8].

The LRINEC score is a diagnostic tool employed in the early diagnosis and risk assessment of NF. It assesses various patient parameters, including C-reactive protein, total white blood cell count, hemoglobin, sodium, creatinine, and glucose levels. LRINEC score exceeding 6 was helpful for the early detection of NF [7]. However, recent validation studies, conducted through retrospective chart reviews, have revealed inconsistent sensitivity in diagnosing NF, ranging from 36% to 83%. A LRINEC score greater than 6 suggests a 50% to 75% likelihood of NF, while an LRINEC score exceeding 8 indicates a greater than 75% probability [5]. 

Based on the initial LRINEC score certain adjustments were implemented to formulate the m-LRINEC scoring system [9]. These modifications include increased levels of High-Sensitive C-reactive protein (HCRP), white blood cell count (WBC), fasting blood glucose, creatinine, and decreased hemoglobin and serum sodium. Additionally, they account for the presence of comorbid diabetes and kidney disease. The outcomes of this modification demonstrated a strong ability to differentiate NF from other severe soft-tissue infections. Specifically, when the cutoff value was set at 17 points, the m-LRINEC scoring system exhibited impressive sensitivity (93.2%) and specificity (86.9%). This corresponded to an area under the curve (AUC) of 0.935 (with a 95% confidence interval of 0.892 to 0.977; p<0.001) [9]. 

Early recognition of NF through the utilization of the LRINEC score or m-LRINEC score enables prompt surgical intervention and administration of broad-spectrum antibiotics, both of which are crucial for successful outcomes. Additionally, continuous monitoring and regular assessments allowed for timely adjustments in treatment plans, ensuring optimal management of organ failure and overall patient care. 

Fortunately, for our patient, the combination of timely surgical intervention and the initiation of broad-spectrum antibiotics, guided by a high clinical suspicion of necrotizing fasciitis and the utilization of the LRINEC score, appeared to be pivotal in achieving a successful outcome. It can be debated whether the limited involvement of muscle or deeper structures played a role in this favorable outcome. Nevertheless, the critical factors contributing to success in the treatment of such patients are the immediate removal of all necrotic tissue, vigilant monitoring, and often, repeat debridement to excise any additional affected and necrotic tissue. Early clinical suspicion, accurate diagnosis, and the indispensable role of a comprehensive, team-based approach in the management of necrotizing fasciitis remain paramount in successfully addressing NF. 

## Conclusions

Necrotizing fasciitis is a dangerous condition that, if left untreated, can cause significant morbidity and potentially mortality. In its initial stages, it can be challenging to distinguish from other superficial skin disorders, such as cellulitis or a hematoma. However, when faced with cases of severe pain, fever, and erythema, physicians should maintain a high level of suspicion and have a low threshold for surgical referral. In cases of uncertainty, utilizing tools such as the LRINEC and m-LRINEC scores can help further distinguish necrotizing versus non-necrotizing soft tissue infections.
